# Threat of drug resistant *Staphylococcus aureus* to health in Nepal

**DOI:** 10.1186/1471-2334-14-157

**Published:** 2014-03-22

**Authors:** Shamshul Ansari, Hari Prasad Nepal, Rajendra Gautam, Nabin Rayamajhi, Sony Shrestha, Goma Upadhyay, Anju Acharya, Moti Lal Chapagain

**Affiliations:** 1 Department of Microbiology, Chitwan Medical College, Bharatpur, Chitwan, Nepal

**Keywords:** *Staphylococcus aureus*, MRSA, Inducible clindamycin resistance, Nepal

## Abstract

**Background:**

*Staphylococcus aureus* is the most commonly isolated organism from the different clinical samples in hospital. The emergence and dissemination of methicillin resistant *Staphylococcus aureus* (MRSA) and growing resistance to non-beta-lactam antibiotics is making treatment of infections due to this organism increasingly difficult.

**Methods:**

This study was conducted to determine the frequency of *Staphylococcus aureus* isolated from different clinical samples, rates of MRSA and full antibiotic susceptibility profiles. Clinical samples were cultured and *Staphylococcus aureus* was identified using standard microbiological methods recommended by the American Society for Microbiology (ASM). Methicillin resistance was confirmed using cefoxitin and oxacillin disks. Inducible clindamycin resistance was identified using D-zone test.

**Results:**

From the processed samples, 306 isolates of *Staphylococcus aureus* were recovered. All the isolates were susceptible to vancomycin and teicoplanin. Methicillin resistance was observed in 43.1% of isolates while inducible clindamycin resistance in 12.4% of the isolates.

**Conclusions:**

The results of our study reveals that rates of resistance to commonly prescribed antibiotics in *Staphylococcus aureus* clinical isolates is high. In particular, rate of methicillin resistance is alarming, prompting concern on the rational use of antibiotics and vigilant laboratory-based surveillance of resistance rates in Nepal.

## Background

*Staphylococcus aureus* is one of the most common human pathogens capable of causing a wide range of infections [1]. Over the past several decades, it has been a leading cause of both hospital and community-acquired infections [2]. It is associated with a variety of clinical infections including septicemia, pneumonia, wound sepsis, septic arthritis, osteomyelitis and post-surgical toxic shock syndrome with substantial rates of morbidity and mortality [3-6].

Increasing rates of antimicrobial resistance, often related to extensive use of antimicrobials, is resulting in fewer treatment options for bacterial infections. This problem is being identified across many different microorganisms, such as *Pseudomonas* spp. and *Enterococcus* spp [7]. Concerningly, rates of resistance to conventional antibiotics in *Staphylococcus aureus* has increased to high levels in some hospitals [8,9]. The incidence of community-acquired and hospital acquired *Staphylococcus aureus* infections has been rising with increasing emergence of drug-resistant strains called methicillin resistant *Staphylococcus aureus* (MRSA) [10-14]. MRSA now represents a global problem. Ever since its isolation, MRSA has emerged as one of the most common causes of hospital acquired infection and continues to remain as an important factor contributing to failure of management [15]. MRSA is frequently resistant to most of the commonly used antimicrobial agents including the aminoglycosides, macrolides, chloramphenicol, tetracycline and fluoroquinolones [16]. In addition, MRSA strains should be considered to be resistant to all cephalosporins, cephems and other beta-lactams (such as ampicillin-sulbactam, amoxicillin-clavulanic acid, ticarcillin-clavulanic acid, piperacillin-tazobactam and the carbapenems) regardless of the *in vitro* test results obtained with those agents [17].

The objectives of this study, therefore, were to identify strains of *Staphylococcus aureus* from clinical samples and determine antimicrobial susceptibility profiles of these isolates.

## Methods

A retrospective study was conducted from December 2010 to December 2012 at Chitwan Medical College Teaching Hospital (a 600 bed teaching hospital), Chitwan, Nepal. Patients were identified and data were extracted using the hospital information and support system.

### Sample collection

The samples were collected in sterile containers by clinicians using aseptic technique and transported to the laboratory without delay. All samples were processed immediately.

### Culture and bacterial identification

For the isolation and identification of *Staphylococcus aureus*, several media used were brain heart infusion (BHI) broth (for blood sample), blood agar (BA), chocolate agar (CHA), MacConkey agar (MA), DNase agar and mannitol salt agar (HiMedia Laboratories Pvt. Limited, India) and the tests used were catalase and coagulase. The collected samples were inoculated onto different culture media. The CHA plates were incubated in a CO_2_ incubator (10% CO_2_) at 37°C for 24 hours. The BA and MA plates were incubated at 37°C for 24 hours in an aerobic atmosphere. *Staphylococcus aureus* was identified by standard microbiological techniques [18]. A purity plate was employed to ensure that the inoculum used for the biochemical tests was pure.

### Antibiotic susceptibility testing

Antibiotic susceptibility tests of the *Staphylococcus aureus* isolates were performed by modified Kirby-Bauer disk diffusion method in compliance with Clinical and Laboratory Standards Institute (CLSI) guidelines using Mueller-Hinton agar standard media. The inhibition zone standards for antimicrobial susceptibility were considered from tables for interpretative zone diameters of CLSI [19]. Antibiotic disks (HiMedia Laboratories, Pvt. Limited, India) used were: penicillin G (10U), ciprofloxacin (5 μg), erythromycin (15 μg), co-trimoxazole (25 μg), gentamicin (10 μg), amikacin (30 μg), cephalexin (30 μg), ceftriaxone 30 μg), cefoxitin (30 μg), oxacillin (1 μg), vancomycin (30 μg), clindamycin (2 μg) and teicoplanin (30 μg). For the identification of MRSA strains, cefoxitin and oxacillin disks were used.

### Identification of methicillin resistant *Staphylococcus aureus* (MRSA) strains

Methicillin resistant *Staphylococcus aureus* (MRSA) was identified by using oxacillin (1 μg) and cefoxitin (30 μg) disks. Plates were incubated at 35°C. Plates containing oxacillin disk were read following a 24 hour incubation period. The diameter of the zone of inhibition (ZOI) of growth was recorded and interpreted as susceptible or resistant according to the criteria of CLSI. *Staphylococcus aureus* isolates were deemed methicillin resistant when the ZOI was ≤10 mm with the oxacillin disk or ≤21 mm with the cefoxitin disk [20].

### Identification of inducible clindamycin resistant strains

Inducible macrolide-lincosamide-streptogramin B (iMLSB) resistance was detected in *Staphylococcus aureus* by Disk approximation test placing a 2 μg clindamycin disk 15 mm away from the edge of a 15 μg erythromycin disk on a MHA plate. Following incubation, organisms that showed flattening of the clindamycin zone adjacent to the erythromycin disk (referred to as a “D” zone) were considered to exhibit inducible clindamycin resistance [20].

*Staphylococcus aureus* ATCC 25923 was used as a control organism for antibiotic sensitivity testing. For MRSA detection, *Staphylococcus aureus* ATCC 25923 and ATCC 43300 were used as negative and positive controls respectively.

### Ethical aspects

The samples used in this study were from routine clinical specimens. Because acquiring the samples did not involve direct patient contact and did not interrupt routine clinical care, consent was not required. Permission to conduct the study was obtained from the Head of the Microbiology Department.

## Results

### Isolate characteristics

A total of 15718 samples (blood 7200, urine 7170, sputum 860 and pus 488) were processed from both in-patients and out-patients. Of 306 isolates of *Staphylococcus aureus* (175 from female patients and 131 from male patients), 120 (39.2%), 83 (27.1%), 63 (20.6%) and 40 (13.1%) were obtained from blood, pus, urine and sputum respectively. Among the processed samples the highest positivity rate was found in pus sample (17.0%) followed by sputum (4.7%), blood (1.7%) and urine (0.9%). The age distribution of the isolates is shown in Table 1.

**Table 1:** **Distribution of clinical isolates of *Staphylococcus aureus *** **according to the age of patients (n = 306)**

**Age of patients in years**	**Number of isolates (%)**
0-10	98 (32.0)
11-20	58 (18.9)
21-30	72 (23.3)
31-40	25 (8.3)
41-50	10(3.4)
51-60	19 (6.3)
61-70	19 (6.3)
71-80	5 (1.5)
**Total**	**306 (100)**

### Antimicrobial susceptibility testing

The antibiogram of the *Staphylococcus aureus* isolates is shown in Table 2. Interestingly, only vancomycin and teicoplanin retained 100% susceptibility. Almost all isolates (94.7%) were resistant to penicillin, and most of them were resistant to other commonly used antibiotics. 43.1% of the isolates were resistant to methicillin by cefoxitin method, and 39.2% were resistant to oxacillin. Of the 100 isolates exhibiting erythromycin resistance, 38 (12.4%) were found to have inducible clindamycin resistance by D-zone test.

**Table 2:** **Antibiogram of *Staphylococcus aureus *** **(n = 306)**

**Antimicrobial agents**	**Resistant isolates (%)**
Penicillin	290 (94.7)
Cotrimoxazole	250 (81.7)
Cephalexin	208 (68.0)
Gentamicin	185 (60.4)
Ciprofloxacin	195 (63.7)
Erythromycin	100 (32.7)
Cefoxitin	132 (43.1)
Oxacillin	120 (39.2)
Clindamycin	84 (27.5)
Amikacin	33 (10.7)
Vancomycin	0
Teicoplanin	0

## Discussion

*Staphylococcus aureus* gain access to the epidermis through cracks in the skin, abrasions, cuts, burns, surgical incisions and intravenous catheters causing wide spectrum of infections, from localized skin lesions such as abscesses, folliculitis to deep seated infections. In the present study, of 306 isolates of *Staphylococcus aureus*, 27% were from pus sample, which signifies their important role in abscess formation.

Antimicrobial resistance has been noticed as one of the paramount microbial threats of the twenty-first century [21]. The multidrug resistance to most of the antibiotics used in infections caused by staphylococci is an increasing problem. The emergence of methicillin resistance among *Staphylococcus aureus* strains led to difficulties in the treatment of infections caused by this organism [22]. Therefore, surveillance on the antimicrobial susceptibility patterns of *Staphylococcus aureus* is of utmost importance in understanding new and emerging resistance trends as well as in the management of both hospital and community-acquired infections.

This study demonstrated that overall rates of susceptibility to commonly prescribed antibiotics in *Staphylococcus aureus* isolates were below 70%, with the exception of clindamycin, amikacin, teicoplanin, and vancomycin. Despite the considerable progress in antimicrobial therapy, resistance in Gram-positive pathogens continues to increase, mainly in relation to the drugs commonly used in medical practice. A high proportion of isolates (94.7%) were resistant to penicillin in this study. This was expected as it has been recognized that only a small proportion of the *Staphylococcus aurues* lineages do not produce beta-lactamases [23-26].

Erythromycin has been used extensively for the treatment of both minor and more serious staphylococcal infections. As a consequence, its role today is increasingly limited due to increasing resistance, which poses a great therapeutic challenge. One third (32.7%) of our isolates were resistant to erythromycin, compared to previous similar studies in Nepal which have round resistance rates of 7.1% in 2010, 11% in 2011, 63.6% in 2013 [24,27,25]. Similarly, trimethoprim-sulfamethoxazole (co-trimoxazole) can be an alternative treatment choice, particularly for non-multi-resistant MRSA infections, although emergence of resistance has been previously observed. This may be due to excessive use of this drug for many other infections and over-the-counter availability of antimicrobials in the developing world for the treatment of many other infections. 81.7% of our isolates were resistant to co-trimoxazole, compared with 42.96% in 2009 [28], 12.5% in 2010 [26], 64% in 2011 [27], and 72.7% in 2013 [25] in Nepal.

While ciprofloxacin is predominantly a Gram-negative drug, it does have activity against *Staphylococcus aureus*. As a consequence of low cost and easy availability, there has been indiscriminate use of ciprofloxacin in Nepal. We identified resistance rate of 63.7%, much higher than previous studies (26% in 2009 [23], 12% in 2011 [27]). The same trend is seen with gentamicin, with 60.4% of our isolates being resistant compared with 46.98% in 2009 [28], 32.73% in 2010 [26], 11% in 2011 [27], and 54.5% in 2013 [25]. Fortunately, rate of resistance to amikacin remains low.

All of our isolates had retained susceptibility to vancomycin and teicoplanin, consistent with previous studies [23,25-27,29], confirming that glycopeptides should be used as empiric therapy for serious staphylococcal infections while waiting for susceptibility testing results to come through [30]. This is likely related to low usage of these agents in Nepal due to high cost. Concerningly, however, lineages with increased resistance to teicoplanin have been observed overseas [31-34].

Clindamycin is one of the drugs of choice in MRSA infections. Macrolide-resistant isolates of *Staphylococcus aureus* may have constitutive or inducible resistance to clindamycin (due to methylation of the 23S rRNA encoded by the erm gene also referred to as MLSB i.e., Macrolide, Lincosamide and type B Streptogramin resistance) or may be resistant only to macrolides (due to efflux mechanism encoded by the msrA gene) [20]. As the presence of an erm gene encoding for inducible resistance may result in treatment failure [35], it is important to perform its testing. We found 38 (12.4%) D-test positive isolates, indicating inducible resistance to clindamycin.

Today the concern of MRSA has reached the pinnacle. It is noteworthy that MRSA can cause both community and hospital acquired infections. Prior antibiotic use is the most common risk factor for colonization and infection with MRSA. In our study, 43.1% of *Staphylococcus aureus* isolates were found to be MRSA which is higher as compared to other studies conducted in Nepal. The incidence of MRSA was reported to be 20% in 2001 [36], 15.4% in 2005 [37], 26.14% in 2008 [29], 39.6% in 2010 [26] and 42.4% in 2013 [25] in Nepal.

Two different methods were employed for the detection of MRSA. The cefoxitin disk method detected 132 (43.1%) MRSA cases while the oxacillin disk method missed 12 cases and detected only 120 (39.2%) *Staphylococcus aureus* as MRSA. According to CLSI [20], the cefoxitin disk test is comparable to the oxacillin disk test for the prediction of mecA-mediated resistance to oxacillin. The cefoxitin disk test is easier to read and thus is the preferred method. Besides, cefoxitin is an inducer of the mecA gene.

There are a number of factors contributing to increasing rates of resistance in *Staphylococcus aureus* in Nepal. Firstly, regulation of antibiotics is poor with their easy and over the counter availability without prescription. Some health care workers and pharmacists are often paid incentives by the pharmaceutical companies to prescribe or sell unnecessary antibiotics. Medical practice by unqualified personnel, who often prescribe unnecessary antibiotics, is yet other common problem in Nepal. Locally produced antibiotics are of questionable quality; and compliance of the patients is also often poor. Many antibiotics are prescribed without culture and sensitivity due to lack of laboratory facilities in most of the areas. Moreover, infection control policies are yet to be instituted properly in most of the hospitals and medical institutions of Nepal.

Thus, regular surveillance of hospital associated infections and antibiotic sensitivity pattern of MRSA; and formulation of definite antibiotic policy may be helpful for reducing the incidence of MRSA infection. Furthermore, healthcare workers should be trained to control hospital infection and infection control program should be conducted effectively in all health care centers.

## Conclusion

This report demonstrates high rates of MRSA and resistance to other drugs in *Staphylococcus aureus* in our hospital. There is a need for longitudinal surveillance of MRSA and its antimicrobial susceptibility profile in Nepal. We recommend effective implementation of hospital infection control and antibiotic policies to control antibiotic resistance in *Staphylococcus aureus.*

## Competing interests

The authors declare that they have no conflict of interest.

## Authors’ contributions

SA, HPN, RG, NR, AA and MLC conceived the design of the study. SA prepared the manuscript with help from HPN, SS and GU. RG, NR, AA and MLC supervised the work and manuscript. All authors read and approved the final manuscript.

## Pre-publication history

The pre-publication history for this paper can be accessed here:

http://www.biomedcentral.com/1471-2334/14/157/prepub

## References

[B1] Foster TJ The *Staphylococcus aureus* “superbug” J Clin Invest 2004 14 1693 1696 10.1172/JCI200423825 15599392 PMC535074

[B2] Lowy FD *Staphylococcus aureus* infection N England J Med 1998 14 520 532 10.1056/NEJM199808203390806 9709046

[B3] Boyce JM Crossley KB, Archer GL Epidemiology and prevention of nosocomial infections The staphylococci in human disease 1997 New York: Churchill Livingstone 309 329

[B4] Shopsin B Kreiswirth BN Molecular epidemiology of methicillin-resistant *Staphylococcus aureus* Emerg Infect Dis 2001 14 323 326 10.3201/eid0702.010236 11294733 PMC2631714

[B5] Cosgrove SE Sakoulas G Perencevich EN Schwaber MJ Karchmer AW Carmeli Y Comparison of mortality associated with methicillin resistant and methicillin sensitive *Staphylococcus aureus* bacteriemia: a meta analysis Clin Infect Dis 2003 14 53 59 10.1086/345476 12491202

[B6] Engemann JJ Carmeli Y Cosgrove SE Fowler VG Bronstein MZ Trivette SL Briggs JP Sexton DJ Kaye KS Adverse clinical and economic outcomes attributable to methicillin resistance among patients with *Staphylococcus aureus* surgical site infection Clin Infect Dis 2003 14 592 598 10.1086/367653 12594640

[B7] Livermore DM Introduction: the challenge of multiresistance Int J Antimicrob 2007 14 51 57 10.1016/j.ijantimicag.2006.09.009 17659208

[B8] Park DW Kim MJ Yang JA Jeong HW Sohn JW Chun BC Risk factors for isolation of low-level mupirocin-resistant versus susceptible methicillin-resistant *Staphylococcus aureus* from patients in intensive care units J Infect 2007 14 337 342 10.1016/j.jinf.2006.06.008 16860870

[B9] Manzur A Vidal M Pujol M Cisnal M Hornero A Masuet C Peña C Gudiol F Aziza J Predictive factors of methicillin resistance among patients with *Staphylococcus aureus* bloodstream infections at hospital admission J Hosp Infect 2007 14 135 141 10.1016/j.jhin.2007.03.015 17513007

[B10] Steinberg JP Clark CC Hackman BO Nosocomial and community acquired *Staphylococcus aureus* bacteremias from 1980 to 1993: impact of intravascular devices and methicillin resistance Clin Infect Dis 1996 14 255 259 10.1093/clinids/23.2.255 8842259

[B11] Emori TG Gaynes RP An overview of nosocomial infections, including the role of the microbiology laboratory Clin Microbiol Rev 1993 14 428 442 8269394 10.1128/cmr.6.4.428 PMC358296

[B12] Deresinski S Methicillin-resistant *Staphylococcus aureus*: an evolutionary, epidemiologic, and therapeutic odyssey Clin Infect Dis 2005 14 562 573 10.1086/427701 15712079

[B13] Fluit AC Wielders CL Verhoef J Schmitz FJ Epidemiology and susceptibility of 3,051 *Staphylococcus aureus* isolates from 25 university hospitals participating in the European SENTRY study J Clin Microbiol 2001 14 3727 3732 10.1128/JCM.39.10.3727-3732.2001 11574603 PMC88419

[B14] Herold BC Immergluck LC Maranan MC Lauderdale DS Gaskin RE Boyle-Vavra S Leitch CD Daum RS Community acquired methicillin-resistant *Staphylococcus aureus* in children with no identified predisposing risk J American Med Assoc 1998 14 593 598 10.1001/jama.279.8.593 9486753

[B15] Salmenlinna S Lyytikainen O Vuopio-Varkila J Community acquired methicillin-resistant *Staphylococcus aureus*, Finland Emerging Infect Dis 2002 14 602 607 10.3201/eid0806.010313 12023917 PMC2738488

[B16] Mandell G Douglas J Bennett R Principles and practice of infectious disease 1995 4 Edinburgh, United Kingdome: Churchill Livingstone Ltd.

[B17] National Committee for Clinical Laboratory Standards (NCCLS) Performance standards for antimicrobial susceptibility testing 2000 7 Wayne PA, USA: NCCLS Vol. 20

[B18] HD I Clinical microbiology procedures handbook 2004 2 ASM press: Washington DC

[B19] Clinical and Laboratory Standard Institute (CLSI) Performance standards for antimicrobial susceptibility testing 2006 Wayne, PA: USA:CLSI: M100-S16

[B20] Clinical and Laboratory Standards Institute (CLSI) Performance standards for antimicrobial susceptibility testing, 17th informational supplement 2007 Wayne, PA: USA:CLSI: M100-S17

[B21] Smolinski MS Hamburg MA Lederberg J Microbial threats to health: emergence, detection and response 2003 Washington: Institute of Medicine 32 25057653

[B22] Waldvogel FA Mandell GL, Bennett JE, Dolin R Staphylococcus aureus (including staphylococcal toxic shock) Mandell, Douglas and Bennett’s principles and practice of infectious diseases 2000 5 Philadelphia, PA: CV, A Harcourt Health Sciences Co

[B23] Tiwari HK Das AK Sapkota D Sivarajan K Pahwa VK Methicillin resistant *Staphylococcus aureus*: prevalence and antibiogram in a tertiary care hospital in western Nepal J Infect Dev Ctries 2009 14 9 681 684 19858569 10.3855/jidc.86

[B24] Shakya B Shrestha S Mitra T Nasal carriage rate of methicillin resistant *Staphylococcus aureus* among at national medical college teaching hospital, Birgunj, Nepal Nepal Med Coll J 2010 14 1 26 29 20677605

[B25] Mishra SK Rijal BP Pokhrel BM Emerging threat of multidrug resistant bugs-*Acinetobacter calcoaceticus baumannii* complex and methicillin resistant *Staphylococcus aureus* BMC Research Notes 2013 14 98 103 10.1186/1756-0500-6-98 23497675 PMC3605284

[B26] Sanjana RK Shah R Chaudhary N Singh YI Prevalence and antimicrobial susceptibility pattern of methicillin-resistant *Staphylococcus aureus* (MRSA) in CMS-teaching hospital: a preliminary report Journal of College of Medical Sciences-Nepal 2010 14 1 1 6

[B27] Baral R Khanal B Acharya A Antimicrobial susceptibility patterns of clinical isolates of *Staphylococcus aureus* in Eastern Nepal Health Renaissance 2011 14 2 78 82

[B28] Shrestha B Pokhrel BM Mohapatra TM Antibiotic susceptibility pattern of nosocomial isolates of *Staphylococcus aureus* in a tertiary care hospital, Nepal J Nepal Med Assoc 2009 14 175 234 238 20795464

[B29] Kumari N Mohapatra TM Singh YI Prevalence of methicillin-resistant *Staphylococcus aureus* (MRSA) in a tertiary-care hospital in Eastern Nepal J Nepal Med Assoc 2008 14 170 53 56 18709031

[B30] Sader HS Gales AC Jones RN Antimicrobial activity of linezolid against gram-positive cocci isolated in Brazil Braz J Infect Dis 2001 14 171 176 10.1590/S1413-86702001000400002 11712961

[B31] Cormican MG Jones RN Emerging resistance to antimicrobial agents in gram-positive bacteria Drugs 1996 14 6 12 10.2165/00003495-199600511-00004 8724811

[B32] Shittu AO Lin J Antimicrobial susceptibility patterns and characterization of clinical isolates of *Staphylococcus aureus* in Kwazulu-Natal province, South Africa BMC Infect Dis 2006 14 125 137 10.1186/1471-2334-6-125 16875502 PMC1564024

[B33] Centers for Disease Control and Prevention *Staphylococcus aureus* resistance to vancomycin in the United States of America MMWR 2002 14 565 56712139181

[B34] Classen M Nouwe J Fang N Ott A Verbrugh H Hofman A Vanbelkum A Uitterlinder A *Staphylococcus aureus* nasal carriage is not associated with known polymorphism in the vitamin D receptor gene FEMS Immunol Med Microbiol 2005 14 173 176 10.1016/j.femsim.2004.08.002 15681147

[B35] Lim HS Lee H Roh KH Yum JH Yong D Lee K Chong Y Prevalence of inducible clindamycin resistance in *Staphylococcal* isolates at a Korean tertiary care hospital Yonsei Med J 2006 14 480 484 10.3349/ymj.2006.47.4.480 16941736 PMC2687727

[B36] Rai CKS Antibiotic susceptibility patters of *Staphylococcus aures* J Nepal MedicalAssociation 2001 14 138

[B37] Subedi S Brahmadarhan KN Antimicrobial susceptibility pattern of clinical isolates of *Staphylococcus aureus* in Nepal Clinical microbiology infect 2005 14 3 253 237 10.1111/j.1469-0691.2004.01056.x 15715723

